# The value of MRI in evaluating the efficacy and complications with the treatment of intra-arterial chemotherapy for retinoblastoma

**DOI:** 10.18632/oncotarget.16423

**Published:** 2017-03-21

**Authors:** Shuxian Chen, Xunda Ji, Ming Liu, Zhengrong Xia, Hui Zheng, Qiufeng Yin, He Wang, Yuhua Li

**Affiliations:** ^1^ Department of Radiology, Xinhua Hospital Affiliated to Shanghai Jiaotong University School of Medicine, Shanghai 200092, China; ^2^ Department of Ophthalmology, Xinhua Hospital Affiliated to Shanghai Jiaotong University School of Medicine, Shanghai 200092, China; ^3^ Institute of Science and Technology for Brain-Inspired Intelligence, Fudan University, Shanghai 200433, China

**Keywords:** retinoblastoma, intra-arterial chemotherapy, MRI, ADC value, children

## Abstract

Retinoblastoma is the most common intraocular malignant tumor of childhood. Intra-arterial chemotherapy (IAC) is a recently popularized technique used for the treatment of retinoblastoma, to decrease mortality, increase preservation of the eye, and prevent blindness. Along with the extensive use of IAC, it is important to apply noninvasive examination methods to assess the activity of the tumor and the risk factors for disease dissemination without histopathological confirmation. There are few studies that have assessed the value of magnetic resonance imaging (MRI) in evaluating the efficacy and complications of IAC for retinoblastoma. We retrospectively analyzed the MRI features of 60 patients with unilateral retinoblastoma given the primary treatment of IAC from January 2014 to February 2016 in our hospital. Our study showed that MRI could well assess the decreased activity of the tumor after IAC, presenting with diminished tumor size, increased apparent diffusion coefficient (ADC) values (from 0.94 ± 0.24 × 10^−3^ mm^2^/s to 2.24 ± 0.40 × 10^−3^ mm^2^/s), and a reduced degree of enhancement of the tumor. Our study also showed that MRI can monitor the risk factors of abnormal enhancement of the postlaminar optic nerve, to avoid unnecessary enucleation. Meanwhile, the results showed that the main late complications after IAC included affected eyeball volume reduction, subretinal hemorrhage, vitreous hemorrhage, vitreous opacity, cataractous len, and choroidal vascular ischemia.

## INTRODUCTION

Retinoblastoma is the most common intraocular malignant tumor of childhood, with a prevalence of one in 15,000–20,000 livebirths [1–5]. Worldwide, the survival of retinoblastoma is significantly variable, with approximately a 30% survival in Africa, 60% in Asia, 80% in Latin American, and 95%–97% in Europe and North America [6].

In the past 10 years, the management of intraocular retinoblastoma has changed dramatically due to the use of intra-arterial chemotherapy (IAC) and intravitreous chemotherapy, contributing to decreased mortality, preservation of the eye, and prevention of blindness [2, 7, 8]. IAC is a recently popularized technique using chemotherapeutic agents delivered directly into the ophthalmic artery. IAC is believed to result in an increased concentration of drug to the tumor, thus saving the eye and reducing systemic side effects [8, 9]. Eyes treated initially with IAC showed an ocular event-free survival percentage at 2 years of > 80%, despite their advanced stage, and IAC achieved a high level of remission in refractory tumors [8, 9]. However, the treatment was associated with significant local side effects [10].

Recently, IAC as a primary therapy has achieved globe preservation and tumor control in a high percentage of advanced stage retinoblastoma of group D and E [8, 10–12]. With the extensive use of a more conservative treatment method, most patients are now treated without histopathological confirmation for assessment of risk factors for disease dissemination and prognosis. Therefore, assessing tumor responses to IAC is crucial in patient treatment. Patients with good responses to IAC have a better disease-free survival rate, improved eye and vision preservation, and a lower risk of recurrence. However, poor responders should be treated aggressively with enucleation to avoid metastases.

Magnetic resonance imaging (MRI) has the advantages of using no harmful radiation and of having high soft tissue resolution. MRI can improve tumor stage diagnoses by providing detailed information, including an evaluation of intraocular tumor extension (i.e., to the choroid, sclera, and/or prelaminar optic nerve) and extraocular (postlaminar or orbital invasion) or intracranial (pineoblastoma or metastases) tumor spread. Few studies have reported the efficacy of treatment with IAC using ophthalmoscopy and ocular US [8, 9], and these did not assess the extraocular (postlaminar or orbital invasion) or intracranial tumor metastases due to poor curative effects. Therefore, MRI could have an important role in follow-up examinations after conservative treatment with IAC to assess the response to therapy, tumor progression, and recurrence, to complement the findings from funduscopy.

Diffusion weighted imaging (DWI) is based on the diffusion of water molecules in tissues, and the tumor water diffusion is associated with tumor cellularity [13–15]. The apparent diffusion coefficient (ADC) of water, as assessed by DWI, could increase after successful treatment, thus reflecting a reduction in the cellular density and a barrier to water motion. Therefore, the DWI and the value of the ADC can be used to distinguish highly cellular regions of tumors from acellular regions, and could be used to monitor the treatment response manifested as changes in cellularity within the tumor. This has been proposed as the technique of choice for detection of responses to treatment in brain tumors, head and neck cancers, and hepatic metastases [13, 14, 16–18].

The purpose of our study was to compare the MRI results of tumors before and after IAC to evaluate the efficacy and side effects of treatment for retinoblastoma.

## RESULTS

### Clinical characteristics

The clinical data of 60 patients (27 females and 33 males) are summarized in Table 1. The mean age at first diagnosis of retinoblastoma was 25.5 months (median, 22.5 months; range, 3–87 months). Of the 60 patients, 26 were right unilateral and 34 were left, and all patients were classified according to the International Classification of Retinoblastoma [4] (ICRB) as group D (*n* = 50; 83.3%) and group E (*n* = 10; 16.7%). The IAC was delivered as the primary therapy in all patients and the secondary therapy of enucleation was performed in six patients. The mean follow-up time was 11 months (range, 6–23 months). The tumors in 59 eyes were stable after treatment, as assessed in the follow-up with funduscopy, and one eye was found with recurrence during the sixth month of follow-up. The stable tumor showed a flat scar, flat or partially calcified remnant, or completely calcified remnant by ophthalmoscopy [19]. There were no significant differences between the patients in groups D and E regarding sex, age, and the side of the affected eyes.

**Table 1:** Clinical characteristics and classification in 60 patients

Clinical characteristics	No. (%)
Mean age(months), median, range	25.5, 22.5, 3–87
Gender	
Female	27 (45)
Male	33 (55)
Laterality (Unilateral)	60 (100)
Eyes undergoing treatment	
Right	26(43.3)
Left	34 (56.7)
Classification of ICRB	
A/B/C	0 (0)
D	50 (83.3)
E	10 (16.7)
Stable tumor after treatment in follow-up with fundus examination	59 (98.3)
Mean follow-up, range (months)	11, 6 to 23
Enucleation after treatment	6 (10)

ICRB = International Classification of Retinoblastoma [4].

### MRI features

The MRI findings for the 60 patients before and after treatment with IAC are summarized in Table 2.

**Table 2:** MRI findings for 60 patients before and after treatment by IAC

MRI features	Before IAC	After IAC	*P* value
Location			
Tumor with totally or partially covering the optic disk	45	3	
Tumor with distance of the tumor to the optic disk less than 2 mm	11	14	< 0.01
Tumor with distance of the tumor to the optic disk more than 2 mm	4	43	
Maximum diameter of tumor (mean values, cm)	1.55 ± 0.26	0.66 ± 0.36	< 0.001
D (*n* = 50)	1.54 ± 0.24	0.61 ± 0.35	< 0.001
E (*n* = 10)	1.60 ± 0.36	0.87 ± 0.38	< 0.001
Thickness (mean values, cm)	1.41 ± 0.34	0.39 ± 0.34	< 0.001
D (*n* = 50)	1.42 ± 0.34	0.34 ± 0.30	< 0.001
E (*n* = 10)	1.36 ± 0.35	0.61 ± 0.45	< 0.001
Maximum cross-sectional area (mean values, cm^2^)	1.51 ± 0.45	0.29 ± 0.23	< 0.001
D (*n* = 50)	1.49 ± 0.44	0.24 ± 0.20	< 0.001
E (*n* = 10)	1.65 ± 0.50	0.53 ± 0.26	< 0.001
ADC (mean values, 10^−3^ mm^2^/s)	0.94 ± 0.24	2.24 ± 0.40	< 0.001
D (*n* = 50)	0.92 ± 0.22	2.24 ± 0.43	< 0.001
E (*n* = 10)	1.00 ± 0.33	2.22 ± 0.27	< 0.001
Enhancement			
Non-enhancement	0	55	
Mild	9	5	< 0.001
Moderate	51	0	
Enhancement of laminar optic nerve, No. (%)	6 (10)	0 (0)	< 0.001

IAC = intra-arterial chemotherapy.

ADC = apparent diffusion coefficient.

All the count data in the table are patient numbers.

### Tumor location

**Figure 1:**
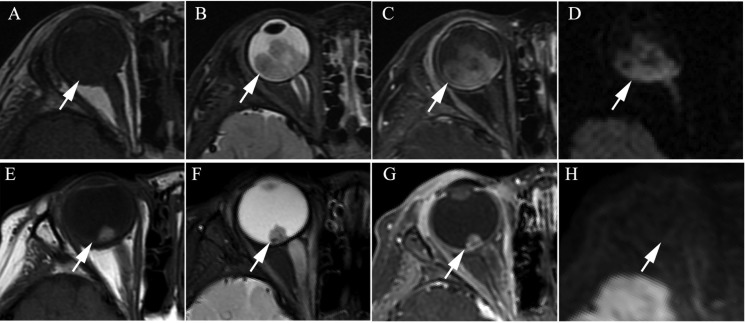
Clinically diagnosed retinoblastoma in a 24-month old male (patient 1) (**A**–**H**) An orbital mass is shown at the right globe with the tumor totally or partially covering the optic disk before intra-arterial chemotherapy (IAC), which demonstrates a slightly higher signal intensity than the ocular fluid as observed in axial T1-weighted magnetic resonance imaging (MRI) (A), low signal intensity (arrow) in the axial T2-weighted MRI (B) and moderate enhancement of the retinoblastoma (arrow) in the axial contrast-enhanced, T1-weighted, fat-saturated MRI (C). The axial DWI (D) shows restricted diffusion (arrow) in the tumor, a finding indicative of high tumor cellularity. (E–H) The axial T2-weighted MRI (F) shows that the size of the tumor is diminished after treatment with IAC and demonstrates a low signal intensity of the residual tumor foci partially covering the optic disk (arrow). The axial contrast-enhanced T1-weighted, fat-saturated MRI (G) shows non-enhancement of the residual tumor foci with comparison to the high signal intensity found in the T1-weighted MRI (E). The axial DWI (H) shows the low signal intensity of the residual tumor foci (arrow).

**Figure 2:**
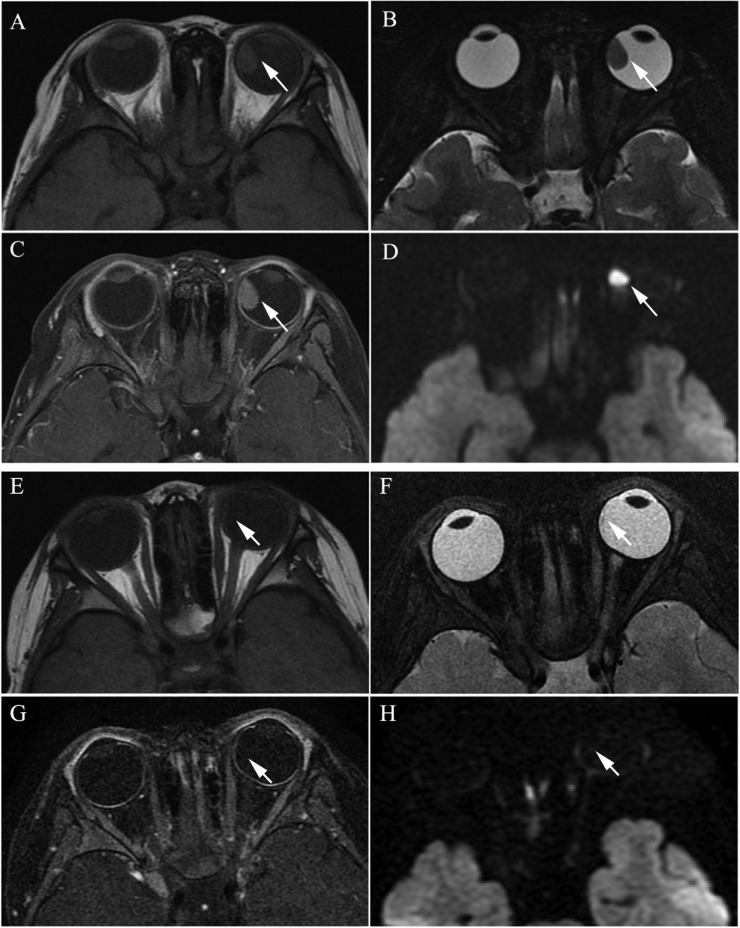
Clinically diagnosed retinoblastoma in a 44-month-old female (patient 2) (**A**–**H**) The orbital origin of the tumor was located in the equator of the left globe with the tumor location far from the optic disk, demonstrating a slightly higher signal intensity than the ocular fluid (arrow) in axial T1-weighted MRI (A), low signal intensity (arrow) in the axial T2-weighted MRI (B) and moderate enhancement of the retinoblastoma (arrow) in the axial contrast-enhanced T1-weighted, fat-saturated MRI (C). The axial DWI (D) shows restricted diffusion (arrow) in the tumor. (E–H) A small amount of the residual tumor foci is shown (arrow) in the axial T2-weighted MRI (F) after three cycles of IAC. The axial contrast-enhanced T1-weighted, fat-saturated MRI (G) shows non-enhancement of the residual tumor foci. The axial DWI (H) shows low signal intensity of the residual tumor foci (arrow).

Of all 60 eyes, the tumors were located completely posterior to the equator in 39 eyes, and involved both the anterior and posterior of the equator in 21 eyes. Before treatment, there were 45 of 60 eyes (75%) with tumors totally or partially covering the optic disks (Figure 1), 11 of 60 eyes (18.3%) with the distance of the tumor to the optic disk < 2 mm, and four of 60 eyes (6.7%) with the distance of the tumor to the optic disk > 2 mm (Figure 2). After IAC, there were only three of 60 eyes (5%) with the tumor totally or partially covering the optic disk, 14 of 60 eyes (23.3%) with the distance of the tumor to the optic disk < 2 mm, and 43 of 60 eyes (71.7%) with the distance of the tumor to the optic disk > 2 mm.

### Tumor sizes

The size of the tumors was obviously diminished after IAC in all 60 eyes, including the mean maximum diameter (*t* = 19.15; *P* < 0.001), the mean thickness (*t* = 18.49; *P* < 0.001), and the maximum cross-sectional area (*t* = 21.35; *P* < 0.001) (Figure 1 and Figure 2). Both group D and group E showed a significant difference in tumor size after IAC. There was no significant difference between the patients of group D and group E in the tumor size before or after treatment.

### Enhancement of the tumor

**Figure 3:**
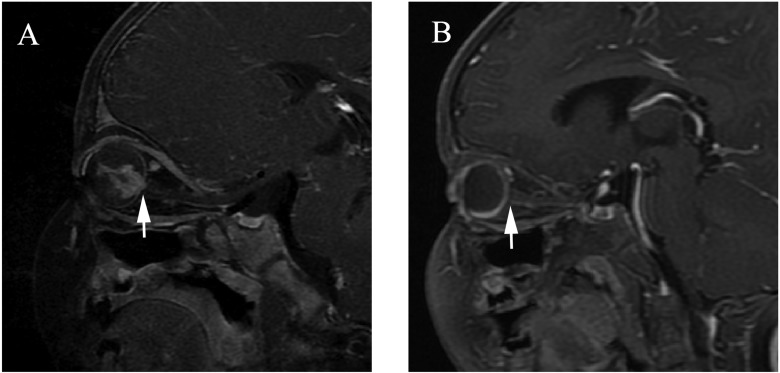
Clinically diagnosed retinoblastoma in a 16-month-old male (patient 3) Oblique sagittal contrast-enhanced T1-weighted, fat-saturated MRI shows abnormal nodular enhancement (arrow) of the postlaminar optic nerve (**A**), a finding indicative of postlaminar optic nerve invasion. After three cycles of intra-arterial chemotherapy (IAC), the abnormal nodular enhancement of the postlaminar optic nerve disappeared (**B**).

Among the 60 eyes, compared to pretreatment, 51 tumors with moderate enhancement decreased to non-enhancement (Figure 1 and Figure 2) (*n* = 46; 90.2%) or slight enhancement (*n* = 5; 9.8%), and the other nine tumors changed from slight enhancement to non-enhancement (*n* = 9; 100%). Nodular enhancement of postlaminar optic nerves observed in six (10%) affected eyes before IAC disappeared after IAC (Figure 3).

### ADC values

**Figure 4:**
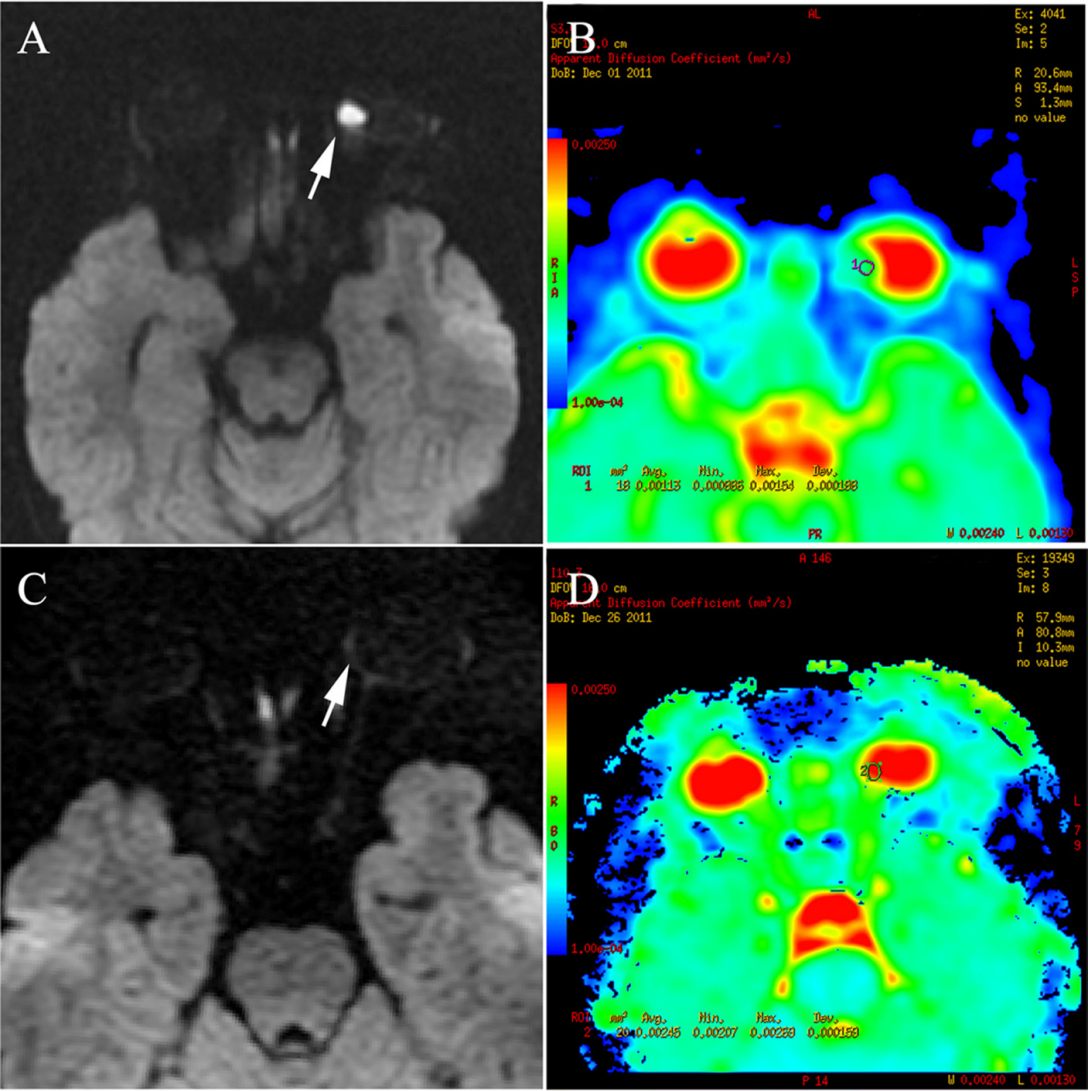
Retinoblastoma of the right eyeball in a 44-month-old female (patient 2) The DWI (**A**) shows hyperintensity in the tumor (arrow), indicating hyper-cellularity. The apparent diffusion coefficient (ADC) of this lesion was 1.13 × 10^−3^ mm^2^/s (**B**). After three cycles of intra-arterial chemotherapy (IAC), DWI shows hypointensity in the tumor region (**C**), with ADC of 2.45 × 10^−3^ mm^2^/s (**D**).

The ADC values of all tumors before IAC were < 1.55 × 10^−3^ mm^2^/s and 90% were < 1.30 × 10^−3^ mm^2^/s (Figure 4). The mean ADC value of the tumors before IAC was 0.94 ± 0.24 × 10^−3^ mm^2^/s. Ninety-two percent (55/60) of the tumors after IAC showed higher ADC values of > 1.90 × 10^−3^ mm^2^/s (Figure 4), with a mean ADC of 2.24 ± 0.40 × 10^−3^ mm^2^/s. Only three of 60 retinoblastoma patients after IAC had an ADC of < 1.55 × 10^−3^ mm^2^/s, which approached the value of the greatest ADC before IAC. The differences in ADC values before and after IAC were significant (*t* = −23.76; *P* < 0.001) as shown in Table 2.

The differences in ADC values of group D or group E were significant (*t* = 21.95; *P* < 0.001 and *t* = 13.5; *P* < 0.001) as is shown in Table 2. However, there was no significant difference between the patients in group D and group E regarding the ADC values before or after treatment (*P* > 0.001).

### Complications

#### Size of eyeballs

The sizes of the eyeballs in the 60 patients before and after treatment with IAC are summarized in Table 3.

**Table 3:** The size of the eyes before and after IAC in 60 patients

Affected eyeball	Before IAC	After IAC	*P* value
Enlargement No.(%)	8 (13.3)	5 (8.3)	< 0.001
Normal No. (%)	43 (71.7)	15 (25.0)	
Shrink No. (%))	9 (15.0)	40 (66.7)	
Affected eyeball			
MCSA (mm^2^)	37.4 *±* 3.9	36.1 *±* 4.3	0.004
AL (mm)	21.1 *±* 1.2	20.3 *±* 2.7	0.022
ED (mm)	21.5 *±* 1.2	20.9 *±* 1.2	< 0.01
Contralateral eyeball			
MCSA (mm^2^)	37.5 *±* 3.5	38.7 *±* 3.1	0.001
AL (mm)	21.2 *±* 1.1	21.4 *±* 0.8	0.017
ED (mm)	21.5 *±* 1.1	21.8 *±* 1.0	0.008

MCSA = maximum cross-sectional area.

AL= axial length.

ED = equatorial diameter.

IAC = intra-arterial chemotherapy.

Before the IAC, of the 60 affected eyeballs, eight eyes (13.3%) showed enlargement, nine eyes (15%) showed shrink, and 43 eyes (71.7%) remained normal in size compared to the contralateral eyeball. After IAC, of the 60 affected eyeballs, five eyes (8.3%) were larger, 40 eyes (66.7%) were smaller, and 15 eyes (25%) remained normal in size compared to the contralateral eyeball.

**Figure 5:**
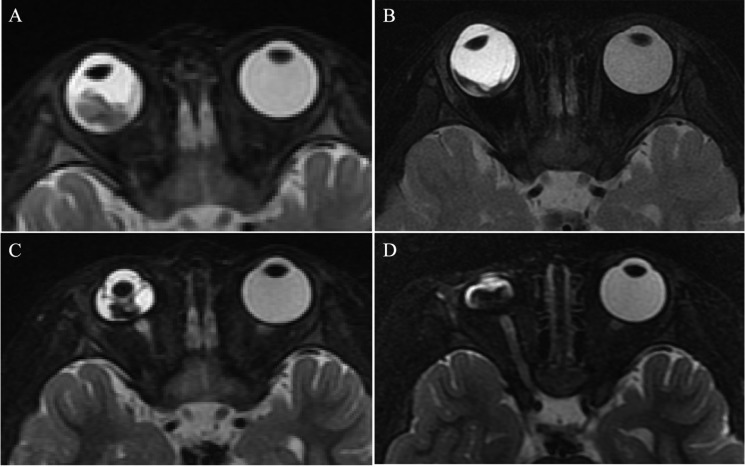
Retinoblastoma diagnosed in a 16-month-old female (patient 4) (**A**–**D**) The axial T2-weighted MRI. The right eyeball with retinoblastoma had a size equal to the contralateral eyeball before treatment (A), and became larger in size than the contralateral eyeball in the second month after three cycles of IAC (B). Eyeball atrophy was found in the fifth month after three cycles of IAC (C) and worsened in the tenth month (D).

The affected eyeballs after IAC had smaller sized eyeballs than did the eyeballs before IAC (Figure 5) and showed significant differences in the maximum cross-sectional area (MCSA) (*t* = 2.60; *P* = 0.004), axial length (AL) (*t* = 2.35; *P* = 0.022), and equatorial diameter (ED) (*t* = 3.90; *P* < 0.001). In comparison, the sizes of the contralateral eyeballs were slightly larger after treatment than before treatment, with significant differences in MCSA (*t* = −3.34; P = 0.001), AL (*t* = −2.46; *P* = 0.017), and ED (*t* = −2.75; P = 0.008). There were no significant differences between the affected eyeball and contralateral eyeball before treatment with MCSA (*t* = −0.142; *P* = 0.887), AL (*t* = −0.496; *P* = 0.622) and ED (*t* = −0.693; *P* = 0.491), however, the size of the affected eyeball was obviously smaller than that of the contralateral eyeball after treatment, with a significant difference in MCSA (*t* = −5.696; *P* < 0.001), AL (t = −2.958; P = 0.004), and ED (*t* = −6.761; *P* < 0.001).

### Other side effects of treatment

The other complications observed by MRI before and after IAC are listed in Table 4, and included retinal detachment with subretinal fluid (16.7% versus 56.7%, respectively), subretinal hemorrhage (5% versus 13.3%, respectively), vitreous hemorrhage (1.7% versus 6.7%, respectively), vitreous opacity (3.3% versus 5%, respectively), cataractous lens (0 versus 6.7%, respectively), extraocular muscle inflammation (0 versus 8.3%, respectively), choroidal vascular ischemia (Figure 6) (0 versus 15%, respectively), and vascular proliferation (0 versus 15%, respectively). Secondary enucleation was performed in six eyes at more than 4 months after IAC. The reasons for enucleation in six eyes included vitreous hemorrhage with cataractous lens (*n* = 1), eyeball atrophy (*n* = 2) (Figure 5), subretinal hemorrhage (*n* = 1), subretinal hemorrhage with eyeball atrophy (*n* = 1), and recurrence with vitreous hemorrhage and cataract (*n* = 1). There was no patient with metastasis and death.

**Table 4:** MRI findings of complications in 60 patients

Complications	Before IAC, No.	After IAC ,No.	*P* value
Retinal detachment with subretinal fluid	10	34	< 0.01
Subretinal hemorrhage	3	8	0.116
Vitreous hemorrhage	1	4	0.173
Vitreous opacity	2	3	0.651
Choroidal vascular ischemia	0	9	0.002
Extraocular muscle inflammation	0	5	0.022
Vascular proliferation	0	9	0.002
Cataract	0	4	0.042
Extraocular invasion or metastasis	0	0	#

IAC = intra-arterial chemotherapy.

# = Unable to be analyzed.

**Figure 6:**
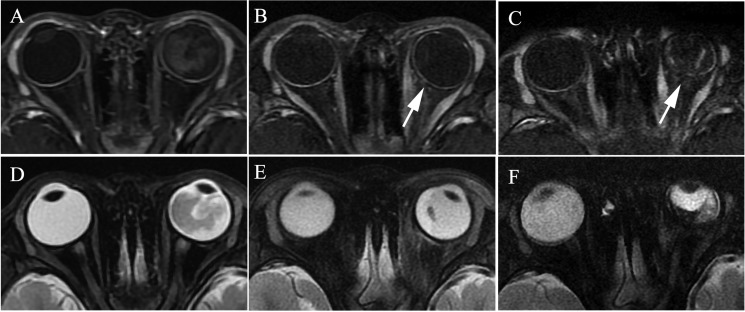
Retinoblastoma of the left eyeball in a 47-month-old male (patient 5) The axial contrast-enhanced T1-weighted, fat-saturated MRI (**A**–**C**) show regular enhancement of the choroid in the left eyeball (A), and decreasing and thinning of the choroid was present when compared with the contralateral eyeball in the first month after two cycles of IAC (B). Intermittent and irregular enhancement of the choroid was present in the fifth month after IAC (C). (**D**–**F**) The axial T2-weighted MRI show a progressive shrinkage of the left globe after IAC.

## DISCUSSION

Retinoblastoma can be a devastating, blinding, and life threatening disease among children, however, it is more curable if diagnosed early [1, 4, 20]. With conservative treatment options significantly improved in the last decades, the main aims in the management of retinoblastoma are globe salvage and reduced systemic toxicity [20]. IAC shows improved tumor control, and is both an effective and safe treatment for retinoblastoma, but it does cause some complications [8–10, 12, 21].

Studies have been reported regarding the efficacy and complications of treatment with IAC using ophthalmoscopy or ocular US [8–11, 22]. However, there are few reported cases using MRI to assess the efficacy and complications of after IAC for retinoblastoma. MRI, with its high tissue contrast and improved spatial resolution, is becoming a primary tool for the diagnosis and staging of retinoblastoma [4, 23]. Post-treatment follow-up with MRI is a rapidly developing discipline that shows promise in assessing efficacy and complications, and it could provide additional information to complement the findings from the funduscopy and the US, especially in the presence of cataract, vitreous hemorrhage, and optic nerve infiltration [23].

In this study, we retrospectively analyzed 60 retinoblastoma patients and compared their MRI characteristics before and after IAC to evaluate the activity of the tumor and the side effects of treatment. Our results showed that primary IAC was an effective treatment for retinoblastoma, with the tumor size diminished in maximum diameter and thickness after treatment by IAC. All of the tumors showed a decreased degree of enhancement, however, five of 60 cases still showed mild enhancement of the tumor. At > 6 months follow-up, using monthly funduscopy, no activity of residual tumor foci was observed.

Optic nerve invasion is one of the primary histopathological risk factors due to its association with a high mortality rate. Surgical treatment is always preferred if there is evidence of optic nerve invasion [4, 23, 24]. Abnormal optic nerve (prelaminar or postlaminar) enhancement is generally regarded as a high risk of optic nerve invasion, especially postlaminar enhancement [4, 24]. The sensitivity and specificity of MRI for depicting postlaminar optic nerve invasion were reported to be approximately 50%−69% and 65%−90%, respectively [4, 23–27]. However, false positive findings of optic nerve enhancement without postlaminar optic nerve invasion have been reported for tumors, causing bulging of the lamina before invading the optic nerve beyond the lamina cribrosa [25, 28], or secondary to inflammation of the optic disc [4, 24], leading to the use of more conservative treatment approaches [29, 30] In our study, six patients with nodular enhancement of the postlaminar optic nerve underwent conservative treatment with IAC rather than enucleation, at the request of their parents. These cases showed significantly deceased nodular enhancements of the postlaminar optic nerve after IAC. We suggest that the drug could have also reached the minimal tumor foci through the blood vessels to the pre- and postlaminar optic nerve. No recurrences and metastases were found in the high-risk patients, with follow-ups of > 8 months, using monthly ophthalmoscopy and quarterly MRI examinations.

Noninvasive DWI has been used to evaluate therapy responses in animal models and humans [16, 31]. Some studies of animal models [31–33] and clinical studies [16, 34] have shown an increase in ADC after variable therapeutic modalities. It is believed that cellular damage leading to necrosis contributed to increased ADCs [35, 36]. In our study, the results showed significant increases in ADCs of all tumors after IAC. Most residual tumor foci showed an ADC > 1.90 × 10^−3^ mm^2^/s, indicating low cell density. There were three cases with ADC values after IAC overlapping the range of the greatest ADC value before IAC. In those cases, the clinical examination and contrast-enhancing T1-weighted imaging data should be weighted appropriately to more accurately determine the activity of the tumor. In our study, one of three cases showed non-enhancement, and the others showed mild enhancement. However, at > 6 months follow-up, using monthly funduscopy, no relapse or recurrence was noted. We suggest that the relatively low ADC value of the residual tumor foci after treatment may be due to the fluid in the retina encapsulating the residual tumor or necrotic tumor tissue due to therapy. A recent study [14] has shown an intermediate ADC value [1.47 (0.99–1.80) × 10^−3^ mm^2^/s] in the necrotic tumor tissue and low ADC values [1.03 (0.72–1.22) ×10^−3^ mm^2^/s] in viable tumor tissues of retinoblastoma patients, further supporting our results.

On the basis of our results, we propose an activity assessment classification scheme for characterizing the activity of the tumor after IAC as follows: (1) non-active, based on an ADC of > 1.90 × 10^−3^ mm^2^/s and non-enhancement of the residual tumor foci; (2) having activity, based on an ADC of < 1.30 × 10^−3^ mm^2^/s with moderate enhancement of the residual tumor foci; and (3) likely activity, for lesions with ADCs of 1.30−1.90 × 10^−3^ mm^2^/s and/or slight enhancement of the residual tumor foci. The optimal clinical examination for patients that showed likely activity was an MRI follow-up at 3 months. The activity assessment classification using MRI can serve as a reference, combined with the findings of the funduscopy, to allow clinicians to decide how to continue treatment. In particular, when a patient has bleeding vitreous hemorrhage and cataract, clinicians can only rely on MRI to evaluate tumor activity.

Despite the advantages regarding tumor control, IAC could also cause persistent complications, including persistent total retinal detachment, subretinal hemorrhage, vitreous hemorrhage, cataract, and retinal or choroidal vascular ischemia. Retinal and choroidal ischemia can be seen on MRI as the lack of choroidal contrast enhancement [23]. The higher risk profile for potential vascular compromise to the eye may cause the delay or arrest developmental of the affected eyes after IAC, and could also cause eyeball atrophy. Media hemorrhage and cataract can lead to blindness and eyeball atrophy would affect the facial appearance, which is ultimately unable to avoid enucleation. Five patients in our study underwent enucleation for hemorrhage, cataract, and/or eyeball atrophy. When media hemorrhage and cataract occurs in the affected eye, contrast-enhancement T1-weight imaging and DWI provides more information than ophthalmoscopy in evaluating the activity of the residual tumor. In our study, one case in group D was diagnosed by MRI as a late relapse with intraocular hemorrhage and cataract at 8 months after IAC. This case was pathologically shown to be an intraocular tumor without extraocular invasion and distant metastasis after enucleation.

The change of size of the affected eyeball was one of the key findings of our study.

Our results showed that there was no statistical difference in the eyeball size between affected eyeballs and contralateral normal eyeballs before IAC. Our results were in disagreement with those of de Graaf et al. [37], that reported that the size of eyeballs with retinoblastoma was smaller than that of normal eyes. Our results showed that the size of affected eyeballs after IAC was smaller than that before IAC, and smaller than that of contralateral eyeballs after IAC. In contrast, with the development of the orbit of children, the size of normal eyeballs was mildly larger than before treatment. We hypothesize that the potential vascular compromise to the eye after IAC could cause the delay or arrest of developmental in the affected eyes. Some patients showed delayed atrophy of the affected eye after more than 9 months follow-up.

However there was insufficient data to obtain statistical information regarding the incidence of atrophy of affected eyes after IAC, because the follow-up time was not long enough and some patients were not followed up by MRI after achieving tumor control. In the future, we will continue to follow these cases to obtain more reliable data.

In conclusion, MRI is a reliable diagnostic approach to evaluate the activity of residual tumor foci after IAC, to monitor the risk factors of optic nerve invasion, extraocular invasion, recurrence, and metastasis and to follow the associated complications of IAC. MRI could be used to monitor the risk factor of abnormal enhancement of the postlaminar optic nerve, thus avoiding unnecessary enucleation.

## MATERIALS AND METHODS

### Patient population

The study was approved by the Ethics Committee of Xinhua Hospital Affiliated to Shanghai Jiao Tong University School of Medicine, Shanghai, China.

Patients were selected from 202 patients with retinoblastoma at our hospital who were treated and followed up in our institution between January 2014 and February 2016. Inclusion criteria were as follows: (1) Clinical and auxiliary examination including ocular US, and MRI proved unilateral retinoblastoma; (2) the MRI was performed before and after treatment by IAC; and (3) the patients were given the primary treatment of IAC, with local chemotherapy as a supplement, including cryotherapy, and laser treatment. Eyes treated primarily with IAC but eventually enucleated were also included. Inclusion criteria were fulfilled by 60 of the patients. MRI reviews after IAC were performed within a mean of 4 months (range, 1–10 months). All patients were followed up for > 6 months (range, 6–23 months) with monthly funduscopy, and MRI was performed from 3–6 months. The patient clinical data were collected at our hospital, including sex, age, affected eye, stage (the ICRB groups), treatment cycles, and follow-up results of ophthalmoscopy.

### Treatment by IAC

IAC was administered under general anesthesia by catheterization of the femoral artery after heparin administration to prevent any embolic complications. A 4FG angiography catheter was passed into the internal carotid artery on the side of the affected eye and flushed continuously with heparinized saline solution. A 1.5FG catheter was placed into the ostium of the ophthalmic artery. Contrast agent was injected to confirm the antegrade flow in the ophthalmic artery. Each chemotherapy dose was diluted in 10 ml of saline and administered in a pulsatile fashion over 10 minutes. Chemotherapeutic medications included melphalan, topotecan and carboplatin. The melphalan dose was 3–7.5 mg, based on the patient age. Topotecan dose was 1mg, and carboplatin dose was 30–50 mg.

Each patient was scheduled for an average of three cycles (range, 2–5 cycles) of IAC at monthly intervals, and the number of cycles was based on the tumor response. Focal cryotherapy or laser therapy was used as needed for tumor consolidation or treatment of recurrence.

### Imaging protocols

MRI was performed under patient sedation on a 3.0-T MR scanner (SignaHdx; GE Medical Systems, Milwaukee, WI, USA) with an eight-channel head coil. Nonenhanced T1-weighted spin-echo images with repetition times (TR)/time of echo (TE) of 400–560/9–14 ms and T2-weighted fast spin-echo images (3000–3700/80–110) were obtained with fat saturation. Images were obtained in at least two planes with a 3 mm slice thickness and a 0.5 mm interslice gap.

Axial contrast-enhanced T1-weighted images with fat saturation (TR/TE of 400 −575/13–15 ms, slice thickness of 2 mm; gap of 0.5 mm; Field of view (FOV) 18–24 cm; matrix 256 × 256) were obtained after intravenous administration of 0.1 mmol/kg of gadopentetate dimeglumine. Oblique sagittal contrast-enhanced T1-weighted imaging with fat saturation, paralleling to the optic nerve, used the same parameters as was used for the axial contrast-enhanced T1-weighted imaging. Axial contrast-enhanced T1-weighted images of the head without fat saturation were obtained with 5 mm section thicknesses and 1 mm intersection gaps.

DWI was acquired in the axial plane prior to administration of contrast medium, by using a single shot echo-planar imaging sequence (TR/TE effective range 3200–5000/70–100 ms; slice thickness of 3 mm; gap of 0.5 mm; FOV 18–24 cm; matrix 128 × 128; excitation at 2). B values of 0 and 1000 s/mm^2^ were applied in three orthogonal directions.

Contrast-enhanced fat-suppressed T1-weighted images (TR/TE of 400 −575/13–15 ms) in axial, coronal, and sagittal oblique (along the course of the optic nerves) planes were then obtained.

All patients underwent MRI as described above.

### Imaging interpretation

The MRI data were evaluated by two observers (YH.L and SX.C, with 25 and 3 years of experience with orbital and brain MRI, respectively). Two observers used a picture archiving and communication system to independently review the MRI data and draw regions of interest on tumors and bilateral eyeballs. Both the T2-weighted images and contrast-enhanced T1-weighted images were used to evaluate the following features: the size of the tumor (maximum diameter, thickness and maximum surface area); distance of the tumor to the optic disk (≥ 2 mm from the optic disk, < 2 mm from the optic disk, or totally or partially covering the optic disk); the optic nerve enhancement (prelaminar or postlaminar) and the enhancement degree of the tumor (non-enhancement, without enhancement; mild enhancement, the enhancement degree was greater than the brain parenchyma, but was less than the extraocular muscles; moderate enhancement, the enhancement degree was comparable to that of the extraocular muscles, but was less than the conjunctiva; obviously enhancement, the enhancement degree was parallel to conjunctiva). T2-weighted MRIs were also used to assess vitreous or subretinal hemorrhage and retinal detachment, and to measure the size of the bilateral eyeballs. The size of the tumor included the maximum diameter (the largest continuous line through the tumor mass on axial contrast-enhanced T1W images),the thickness (the maximum longitudinal diameter of the tumor on coronal or sagittal contrast-enhanced T1W images) and the maximum surface area (the largest sectional area of the tumor on axial contrast-enhanced T1W images). The MCSA, AL, and ED on T2 weighted images were used to represent the size of the eyeballs in order to compare the changes of eyeball size before and after treatment. MCSA was defined as the maximum cross-sectional area of the eye outline, with the boundary in the scleral outer edge. AL was defined as the distance between the anterior corneal surface and inner surfaces of the sclera, and was measured in the optic axis, which is a line that passes through the center of the lens, perpendicular to its anterior and posterior surfaces. ED was defined as the maximum distance between the inner surfaces of the sclera, perpendicular to the AL. The “enlargement” was defined as the size of the affected eyeball was > 5% for MCSA and > 1 mm for AL or ED than the contralateral eyeball and the “shrink” was defined as the size of the affected eyeball was < 5% for MCSA and < 1 mm for AL or ED than the contralateral eyeball.

We combined the results of the data obtained by the two observers by calculating the size of the tumors and eyeballs when the difference between the two readers was < 5% for a maximum surface area and < 1 mm for the diameter. If the difference between the two observers was > 5%, the consensus diameters or maximum surface area were determined by obtaining measurements together. Discrepancies in interpretation were resolved by consensus.

The ADC measurements were performed by one radiologist, on a workstation (ADW4.2, GE Medical Systems). On the ADC maps, three circular regions of interest (ROIs) were placed at targeted areas in the tumor, and the targeted areas in the tumor after treatment were located through T2-weighted imaging. When the residual tumor is too small, the ROI were located in the area of the original tumor. The final ADC values of each patient were determined by the average results of all measured ROIs.

### Statistical analysis

All data were analyzed with the Statistical Package for the Social Sciences (SPSS) software package, version 18.0 (SPSS, Chicago, IL, USA). Paired samples *t*-tests were used to assess pair-wise comparisons of quantitative MRI data (tumor size, ADC values, and the size of eyeballs following treatment). Independent sample *t*-tests were used to compare the tumor size, including the MSCA, AL, and ED before or after treatment. Chi-square test were used to analyzed the count data. Statistical significance was defined as *P* values < 0.05.
